# Long-Term Effect of Interferon Plus Ribavirin on Hepatitis B Surface Antigen Seroclearance in Patients Dually Infected with Hepatitis B and C Viruses

**DOI:** 10.1371/journal.pone.0020752

**Published:** 2011-06-13

**Authors:** Ming-Lun Yeh, Chao-Hung Hung, Jee-Fu Huang, Chun-Jen Liu, Chuan-Mo Lee, Chia-Yen Dai, Jing-Houng Wang, Zu-Yau Lin, Sheng-Nan Lu, Tsung-Hui Hu, Ming-Lung Yu, Jia-Horng Kao, Wan-Long Chuang, Pei-Jer Chen, Ding-Shinn Chen

**Affiliations:** 1 Hepatobiliary Division, Department of Internal Medicine, Kaohsiung Medical University Hospital, Kaohsiung, Taiwan; 2 Faculty of Internal Medicine, College of Medicine, Kaohsiung Medical University, Kaohsiung, Taiwan; 3 Division of Hepato-Gastroenterology, Department of Internal Medicine, Kaohsiung Chang Gung Memorial Hospital, Chang Gung University College of Medicine, Kaohsiung, Taiwan; 4 Department of Internal Medicine, Kaohsiung Municipal Hsiao-Kang Hospital, Kaohsiung Medical University, Kaohsiung, Taiwan; 5 Department of Internal Medicine, National Taiwan University College of Medicine and National Taiwan University Hospital, Taipei, Taiwan; 6 Department of Internal Medicine, Kaohsiung Municipal Ta-Tung Hospital, Kaohsiung Medical University Hospital, Kaohsiung Medical University, Kaohsiung, Taiwan; The University of Hong Kong, Hong Kong

## Abstract

**Background:**

Interferon-α/ribavirin combination therapy might promote hepatitis B surface antigen (HBsAg) seroclearance in patients dually infected with hepatitis B and C viruses (HBV/HCV), but the long-term effect remains unclear. We aimed to investigate the rate of and the factors associated with HBsAg seroclearance during long-term follow-up after interferon-α/ribavirin combination therapy in HBV/HCV dually-infected patients.

**Methodology/Principal Findings:**

Eighty-one patients who received interferon-α/ribavirin combination therapy for 24 weeks with a follow-up period of >24 weeks were enrolled. HBV serological markers and HBV DNA were determined every 6 months. Early and late HBsAg seroclearance were defined as HBsAg loss in less or more than 6 months after end-of-treatment, respectively. Fifteen (18.5%) patients had HBsAg seroclearance during a mean follow-up period of 3.4 (0.5–5.1) years. The 5-year cumulative incidence was 25.6%. Baseline cirrhosis and HBV DNA negativity 1 year after end-of-treatment were independently predictive of HBsAg seroclearance with an odds ratio (OR), 95% confidence intervals (CI) of 16.6, 1.8–153 and 9.2, 1.4–62.1, respectively, by Cox regression hazard analysis. Four patients developed early and 11 developed late HBsAg seroclearance, respectively. Cox regression hazard analysis showed no factor was associated with early HBsAg seroclearance, whilst HBV DNA negativity 1 year after end-of-treatment was the only significant factor predicting late HBsAg loss (OR, 43.0; CI, 2.5–745). Five patients had HBsAg seroconversion with a 5-year cumulative incidence of 8.3%. HBV DNA negativity at baseline and one year after EOT had a trend for HBsAg seroconversion. HCV response did not correlate to HBsAg loss.

**Conclusions:**

We demonstrated that interferon-α/ribavirin had long-term effect on HBsAg seroclearance in dually HBV/HCV-infected patients. Baseline cirrhosis and seroclearance of HBV DNA 1 year after end-of-treatment were significant factors associated with HBsAg seroclearance.

## Introduction

Hepatitis B virus (HBV) and hepatitis C virus (HCV) infections are the two leading causes of chronic liver disease, liver cirrhosis (LC), and hepatocellular carcinoma (HCC) [1], [2]. Since both viruses share the same transmission routes, dual HBV/HCV infection is not uncommon, accounting for 10–40% of patients with chronic hepatitis in Taiwan and Europe [3], [4]. Previous studies also indicated that HBV/HCV dually infected patients had a much higher risk of developing LC or HCC than those with HBV or HCV infection alone [5], [6]

Seroclearance of hepatitis B surface antigen (HBsAg) is a rare event in chronic hepatitis B (CHB) patients [7], [8]. The estimated annual incidence of HBsAg seroclearance is 0.1–2.26% [9], [10]. A recent study demonstrated the annual HBsAg seroclearance rate has quite differed in Hepatitis B e antigen (HBeAg) positive and negative populations (0.39 versus 2.61 per 100 person-years, P<0.001) [10]. HBsAg seroclearance usually confers a favorable outcome and is the optimal treatment goal. However, HBsAg seroclearance is very rarely observed in HBV infected patients receiving current antiviral agents, with an annual rate of 2.4–3.2% with interferon (IFN) or pegylated IFN therapy and only <1% with nucleoside/nucleotide analogues [11], [12]

IFN is an antiviral agent approved for the treatment of CHB and chronic hepatitis C. Although IFN monotherapy has been ineffective in treating HBV/HCV dually infected patients [13], the introduction of ribavirin in combination with IFN-α or pegylated IFN-α for HBV/HCV dually infected patients has been shown to be as effective for patients with HCV monoinfection in terms of sustained eradication of HCV viremia [14], [15]. Interestingly, the combination therapy could also promote HBsAg seroclearance in HBV/HCV dually infected patients, which can reach 11.2% 6 months after end of treatment [16], [17], [18], [19], [20]. Recently, an increased rate of HBsAg clearance to 8.7% was observed in patients with HBeAg-negative chronic hepatitis B after 3 years of peginterferon alpha-2a as a first-line treatment [21]. Whether the rate of HBsAg loss among HBV/HCV dually infected patient increases overtime after IFN plus ribavirin combination therapy has not been well established. We conducted a long-term follow-up study to explore the cumulative rate of and predictive factors associated with HBsAg seroclearance in HBV/HCV dually infected patients after IFN plus ribavirin combination therapy.

## Materials and Methods

### Patients

The present study is based on three previous studies investigating the efficacy of IFN-α/ribavirin combination therapy in HBV/HCV dually infected patients [16], [17], [18]. From April 1999 to April 2002, 102 patients with dual HBV/HCV infections were enrolled into the three studies. All the patients were seropositive for HCV antibody (anti-HCV) and HBsAg for more than 6 months. Except three patients, all the others were positive for serum HCV RNA (detect by a standardized qualitative polymerase chain reaction (PCR) assay (Cobas Amplicor HCV Monitor Version 2.0; Roche Diagnostics, Branchburg, NJ, USA; detection limit 50 IU/ml)). Patients with human immunodeficiency virus or hepatitis delta virus infection, autoimmune hepatitis, primary biliary cirrhosis, sclerosing cholangitis, Wilson's disease, α_1_-antitrypsin deficiency, decompensated cirrhosis, overt hepatic failure, a current or past history of alcohol abuse (≥80 gm ethanol per day), a psychiatric condition, or evidence of HCC were excluded.

All patients received a regimen of 3–6 MU IFN-α thrice weekly plus ribavirin 800–1200 mg per-day for 24 weeks. Of the 102 patients, 81 who were followed up more than 24 weeks after end of treatment (EOT) were enrolled into the present long-term follow-up study. During the follow-up period, liver function tests, HBV serological markers and HBV DNA were tested at an interval of 6 months. HBsAg, antibody to HBsAg (anti-HBs), hepatitis B e antigen (HBeAg), antibody to HBeAg (anti-HBe), and anti-HCV were detected with commercially available enzyme-linked immunosorbent assay kits (Abbott Laboratories, North Chicago, IL, USA) in each local laboratory unit. Serum HBV DNA was determined by a standardized automated quantitative PCR assay (Cobas Amplicor HBV Monitor; Roche Diagnostics; detection limit 200 copies/ml). Serum HCV RNA levels were measured using the branched DNA assay (VERSANT HCV-RNA 3.0. Assay, Bayer Diagnostics, Emeryville, CA, USA; quantification limit 615 IU/ml). The study was conducted according to the guidelines of the Declaration of Helsinki, with the principles of good clinical practice, and was approved by local ethics committees for patient's chart review and data analysis without informed consent.

### Study endpoint

The primary aim of the study was the loss of serum HBsAg in HBV/HCV dually infected patients after IFN-α/ribavirin combination therapy. The secondary aim was to evaluate the factors related to HBsAg loss.

### Definitions

HCV sustained virological response (SVR) was defined as undetectable HCV RNA at 24 weeks after EOT. We defined early HBsAg seroclearance as HBsAg loss within 6 months after EOT and late HBsAg seroclearance as HBsAg loss beyond 6 months after EOT. HBsAg seroconversion was defined as HBsAg loss and seropositive anti-HBs.

### Statistical analysis

Means and standard deviations were used to describe the distribution of continuous variables. Independent *t*-tests were used for comparing continuous variables. Chi-square and Fisher's exact test were used for comparing categorical variables. Cox proportional hazards regression models were used to identify the independent factors that might influence HBsAg seroclearance. The cumulative probability of HBsAg seroclearance was analyzed by the Kaplan-Meier actuarial curve method with the log-rank test. All tests were two-sided, and a *P* value of <0.05 was considered statistically significant. All analyses were performed using the SPSS 16.0 statistical package (SPSS, Inc., Chicago, IL, USA).

## Results

### Patient demographics

Table 1 shows baseline and follow-up data of the 81 patients. The mean post-treatment follow-up period was 3.4±1.1 (0.5–5.1) years. Four (4.9%) patients were seropositive for HBeAg and 72 (88.9%) were seropositive for anti-HBe at baseline. Thirty-four (42%) patients had undetectable HBV DNA at baseline. Baseline HCV RNA level was 5.5±1.4 log IU/ml. Fifty-nine (72.8%) patients achieved an HCV SVR after combination therapy. During the follow-up period, 47 (58%), 36 (44.4%), and 26 (40%) patients had undetectable HBV DNA at EOT and 6 and 12 months after EOT, respectively.

**Table 1:** Baseline and follow-up data.

Baseline data	
Male, n (%)	56 (69.1)
Mean age (years), mean ± SD	46.4±11.8
Post-treatment follow-up period, year, mean ± SD (range)	3.7±1.2 (0.5–4.9)
Baseline ALT level (U/L), mean ± SD	112.9±77.8
Liver cirrhosis, n (%)	7 (8.6)
Positive HBeAg, n (%)	4 (4.9)
Baseline HBV DNA negativity, n (%)	34 (42)
Baseline HCV RNA level (log IU/ml), mean ± SD	5.5±1.4

Follow-up data	
Achievement of an HCV SVR, n (%)	59 (72.8)
HBV DNA negativity at EOT, n (%)	47 (58)
HBV DNA negativity 6 months after EOT, n (%)	36 (44.4)
HBV DNA negativity 1 year after EOT, n/N (%)	26/65 (40)

SD, standard deviation; ALT, alanine aminotransferase; HBeAg, hepatitis B e antigen; HBV, hepatitis B virus; HCV, hepatitis C virus; SVR, sustained virologic response; EOT, end of treatment.

### Cumulative probability and predictive factors of HBsAg seroclearance

**Figure 1:**
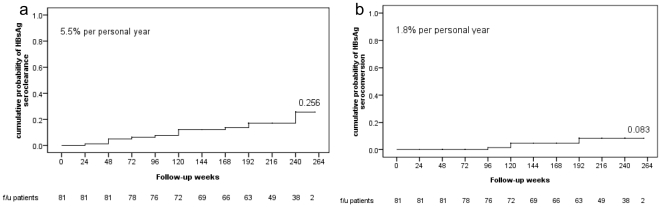
5-year cumulative probability of HBsAg seroclearance (a) and seroconversion (b) in HBV/HCV dually infected patients. Fifteen patients had HBsAg seroclearance during the follow-up period with a 5-year cumulative incidence of 25.6% (a). HBsAg seroconversion was found in 5 of the 81 patients. The 5-year cumulative incidence was 8.3% (b). HBsAg, hepatitis B surface antigen; HBV, hepatitis B virus; HCV, hepatitis C virus.

**Figure 2:**
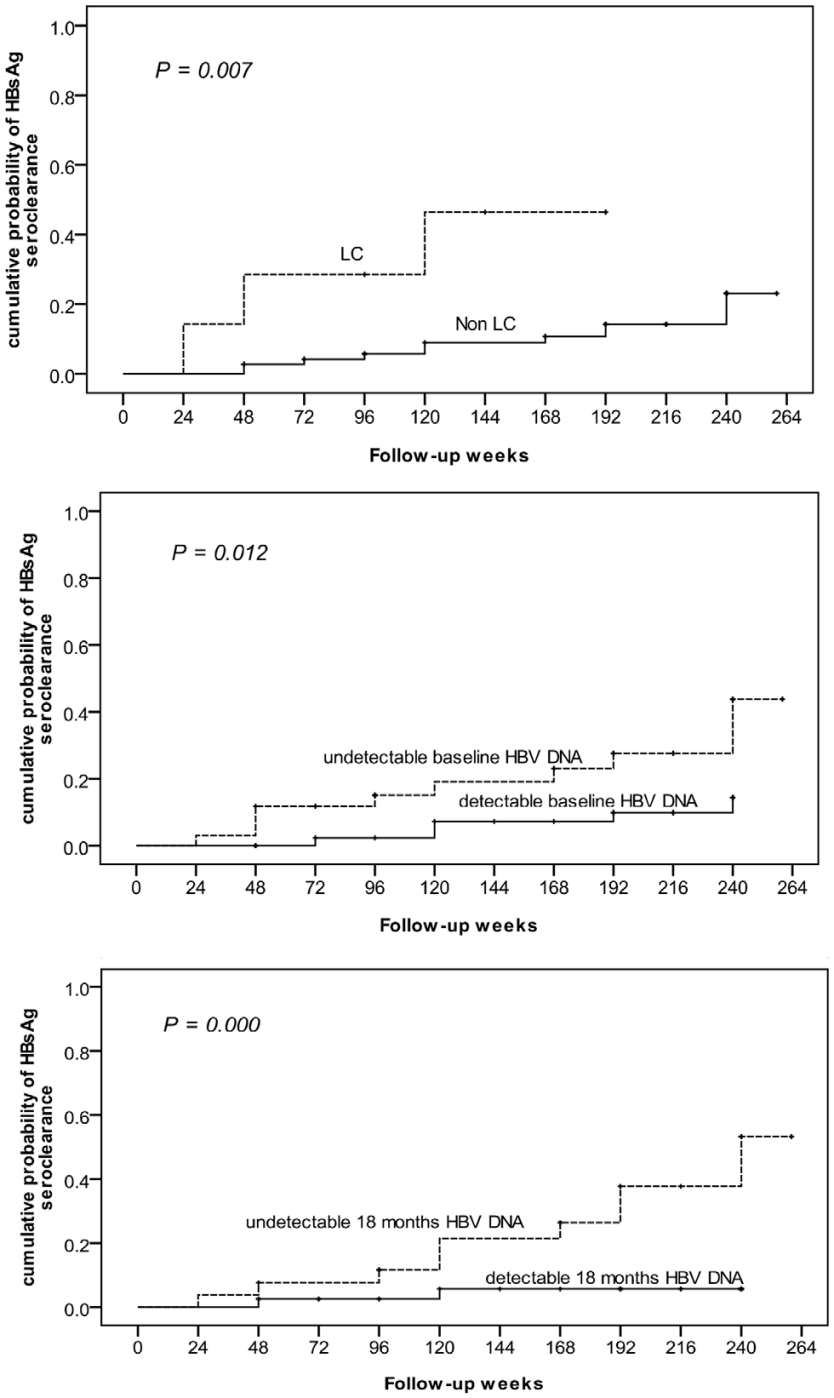
5-year cumulative probability of HBsAg seroclearance was significantly associated with LC and HBV DNA. Using the Kaplan-Meier method, the 5-year cumulative probability of HBsAg seroclearance was significantly associated with LC (*P* = 0.007), baseline HBV DNA negativity (*P* = 0.012) and HBV DNA negativity 1 year after EOT (*P* = 0.000). HBsAg, hepatitis B surface antigen; LC, liver cirrhosis; HBV, hepatitis B virus; EOT, end of treatment.

Fifteen patients had HBsAg seroclearance during the follow-up period with a 5-year cumulative incidence of 25.6% (Figure 1a). The annual incidence was 5.5% per person year. We analyzed baseline and follow-up factors associated with HBsAg seroclearance (Table 2). Baseline HBV DNA negativity (*P* = 0.043) and HBV DNA negativity 1 year after EOT (*P* = 0.002) were significantly positive factors associated with HBsAg seroclearance in univariate analysis. HBsAg seroclearance developed among 29.4% (10/34) of patients with baseline HBV DNA negativity and 38.5% (10/26) of patients with HBV DNA negativity 1 year after EOT, which was significantly higher than the 10.6% (5/47) among patients with baseline HBV DNA positivity (p = 0.043) and the 5.1% (2/39) among patients with HBV DNA positivity 1 year after EOT (P = 0.002), respectively. Baseline HCV RNA level and HCV SVR achievement were not associated with HBsAg seroclearance. Using the Kaplan-Meier method, we observed that the cumulative probability of HBsAg seroclearance was significantly associated with LC (*P* = 0.007), baseline HBV DNA negativity (*P* = 0.012) and HBV DNA negativity 1 year after EOT (*P* = 0.000, Figure 2). In multivariate analysis by using Cox regression hazard analysis, LC (*P* = 0.013; odds ratio = 16.6; 95% CI = 1.8–153.0) and HBV DNA negativity 1 year after EOT (*P* = 0.023; odds ratio = 9.2; 95% CI = 1.4–62.1) were the independent factors associated with HBsAg seroclearance.

**Table 2:** Comparison of baseline and follow-up factors in HBV/HCV dually infected patients who did versus did not achieve HBsAg seroclearance.

	HBsAg seroclearance		P value
	Yes	No	
Number of patients	15	66	
Male, n (%)	10 (66.7)	46 (69.7)	1.000
Age (years)	51.5±9.2	45.2±12.1	0.064
Posttreatment follow-up period, year, Mean±SD (range)	3.3±1.4 (0.5–4.5)	3.7±1.1 (0.5–4.9)	0.274
Baseline ALT level (U/L)	136.6±78.3	107.5±77.3	0.193
Liver cirrhosis, n (%)	3 (20)	4 (6.1)	0.114
Positive HBeAg, n (%)	0 (0)	4 (6.1)	1.000
Baseline HBV DNA negativity, n (%)	10 (66.7)	24 (36.4)	0.043
Baseline HCV RNA level (log IU/ml)	5.7±1.1	5.4±1.5	0.502
Achievement of an HCV SVR, n (%)	10 (66.7)	49 (74.2)	0.537
HBV DNA negativity EOT, n (%)	11 (73.3)	36 (54.5)	0.250
HBV DNA negativity 6 months after EOT, n (%)	9 (60)	27 (40.9)	0.251
HBV DNA negativity 1 year after EOT, n/N (%)	10/12 (83.3)	16/53 (30.2)	0.002

HBsAg, hepatitis B s antigen; SD, standard deviation; ALT, alanine aminotransferase; HBeAg, hepatitis B e antigen; HBV, hepatitis B virus; HCV, hepatitis C virus; SVR, sustained virologic response; EOT, end of treatment.

We also compare the long-term HBsAg seroclearance rate in our conventional interferon group to our another cohort using Pegylated-Interferon (Peg-IFN) in HBV/HCV co-infected patients. During 3.5 years post-treat follow up period, 12 of 69 patients had HBsAg seroclearance in our cohort and 12 of 36 patients had HBsAg seroclearance in Peg-IFN cohort. There is no significant difference between the two groups (Chi-Square test, P value = 0.087).

### Early and late HBsAg seroclearance

**Figure 3:**
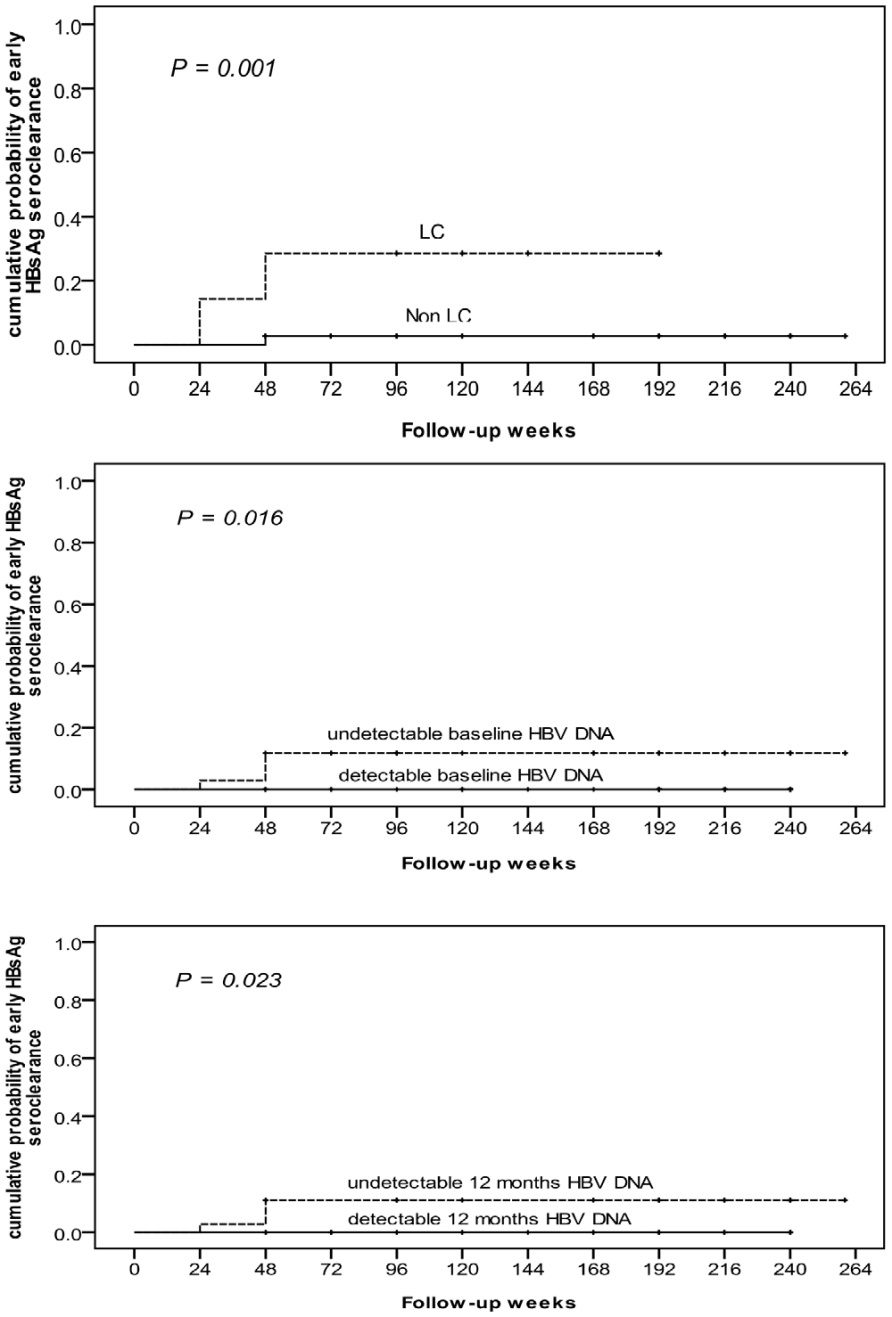
Cumulative probability of early HBsAg seroclearance was significantly associated with LC and HBV DNA. Using Kaplan-Meier method, the cumulative probability of early HBsAg seroclearance remained significantly associated with LC, HBV DNA negativity at baseline and 6 months after EOT (*P* = 0.001, 0.016, 0.023). HBsAg, hepatitis B surface antigen; LC, liver cirrhosis; HBV, hepatitis B virus; EOT, end of treatment.

Of the 15 patients who achieved HBsAg seroclearance, 4 showed seroclearance in less than 6 months after EOT. In univariate analysis, LC, baseline HBV DNA negativity and HBV DNA negativity 6 months after EOT were significantly associated with early HBsAg seroclearance (*P* = 0.036, 0.028, 0.035, respectively, Table 3). By using Kaplan-Meier method, the cumulative probability of early HBsAg seroclearance remained significantly associated with LC, HBV DNA negativity at baseline and 6 months after EOT (*P* = 0.001, 0.016, 0.023, respectively, Figure 3). Nevertheless, in multivariate analysis by using Cox regression hazard analysis, no factor was independently associated with the development of early HBsAg seroclearance.

**Table 3:** Comparison of baseline and follow-up factors of HBV/HCV dually infected patients who did versus did not achieve early/late HBsAg seroclearance.

	Early HBsAg seroclearance		P value	Late HBsAg seroclearance		P value
	Yes	No		Yes	No	
Number of patients	4	77		11	66	
Male, n (%)	2 (50)	54 (70.1)	0.583	8 (72.7)	46 (69.7)	1.000
Age (years)	56.0±7.3	45.9±11.8	0.095	49.8±9.6	45.2±12.1	0.234
Post-treatment follow-up period, year, Mean±SD (range)	3.9±0.9 (2.5–4.5)	3.7±1.2 (0.5–4.9)	0.711	3.1±1.6 (1.0–4.5)	3.7±1.1 (0.5–4.9)	0.209
Baseline ALT level (U/L)	168.5±64.2	110.0±77.7	0.144	125.0±82.4	107.5±77.3	0.494
Liver cirrhosis, n (%)	2 (50)	5 (6.5)	0.036	1 (9.1)	4 (6.1)	0.548
Positive HBeAg, n (%)	0 (0)	4 (5.2)	1.000	0 (0)	4 (6.1)	1.000
Baseline HBV DNA negativity, n (%)	4 (100)	30 (39)	0.028	6 (54.5)	24 (36.4)	0.322
Baseline HCV RNA level (log IU/ml)	6.4±0.7	5.4±1.4	0.197	5.5±1.1	5.4±1.5	0.949
Achievement of an HCV SVR, n (%)	3 (75)	56 (72.7)	1.000	7 (63.6)	49 (74.2)	0.479
HBV DNA negativity EOT, n (%)	3 (75)	44 (57.1)	0.635	8 (72.7)	36 (54.5)	0.335
HBV DNA negativity 6 months after EOT, n (%)	4 (100)	32 (41.6)	0.035	5 (45.5)	27 (40.9)	1.000
HBV DNA negativity 1 year after EOT, n/N (%)				8/9 (88.9%)	16/53 (30.2)	0.001

HBsAg, hepatitis B s antigen; SD, standard deviation; ALT, alanine aminotransferase; HBeAg, hepatitis B e antigen; HBV, hepatitis B virus; HCV, hepatitis C virus; SVR, sustained virologic response; EOT, end of treatment.

**Figure 4:**
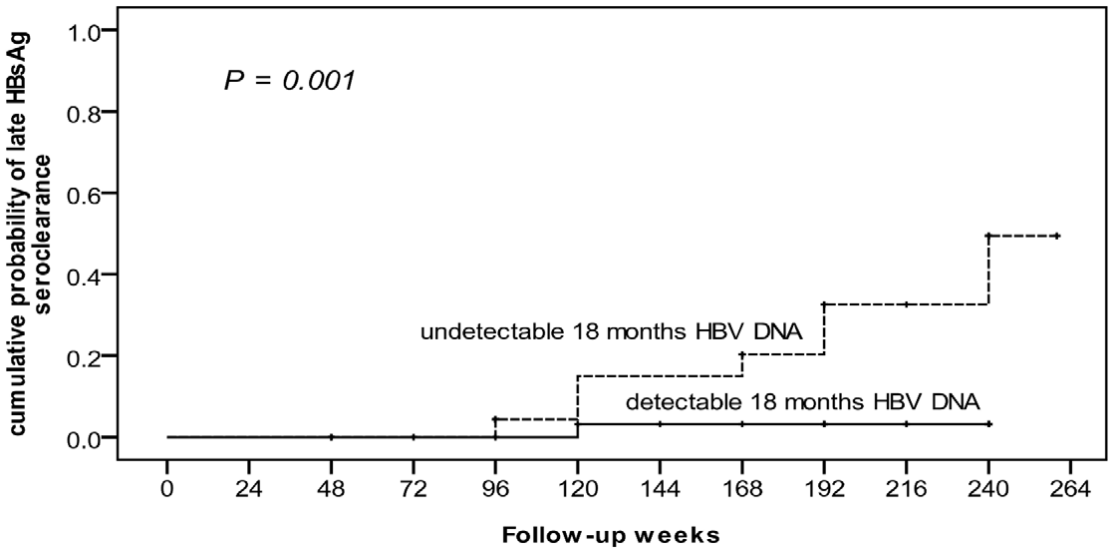
Cumulative probability of late HBsAg seroclearance was significantly associated with HBV DNA. Using Kaplan-Meier method, the cumulative probability of late HBsAg seroclearance was significantly associated with HBV DNA negativity 1 year after EOT (*P* = 0.001). HBsAg, hepatitis B surface antigen; HBV, hepatitis B virus; EOT, end of treatment.

Eleven of the remaining 77 patients achieved HBsAg seroclearance beyond 6 months after EOT. In univariate analysis, HBV DNA negativity 1 year after EOT was the only significant factor related to late HBsAg seroclearance (*P* = 0.001, Table 3). By using Kaplan-Meier method, the cumulative probability of late HBsAg seroclearance was significantly associated with HBV DNA negativity 1 year after EOT (*P* = 0.001, Figure 4). Cox regression hazard analysis showed HBV DNA negativity 1 year after EOT was the only significant factor related to late HBsAg seroclearance (*P* = 0.010; odds ratio = 43.0; 95% CI = 2.5–745).

Comparing the three cohort groups of early, late and non-HBsAg loss, we found a trend of LC and baseline HBV DNA negativity for the overall three groups (P = 0.012, 0.011).

### Cumulative probability and predictive factors of HBsAg seroconversion

HBsAg seroconversion was found in 5 of the 81 patients. One patient achieved seroconversion by 18 months, two by 24 months, and two by 42 months after EOT. The 5-year cumulative incidence was 8.3% (Figure 1b). The annual incidence was 1.8% per person year. All five patients were HBeAg seronegative and anti-HBe seropositive at baseline. Comparing the three groups of HBsAg seroconversion, HBsAg seroclearance without seroconversion and non-HBsAg seroclearance, there was a trend of baseline HBV DNA negativity and HBV DNA negativity one year after EOT (P = 0.032, 0.004) (Table 4).

**Table 4:** Comparison of baseline and follow-up factors in HBV/HCV dually infected patients who did achieve HBsAg seroconversion versus HBsAg seroclearance without seroconversion and non-HBsAg seroclearance.

	HBsAg seroclearance with seroconversion		Non-HBsAg seroclearance	P value
	Yes	No		
Number of patients	5	10	66	
Male, n (%)	2 (40)	8 (80)	46 (69.7)	0.801
Age (years)	49.4±10.4	52.5±9.0	45.2±12.1	0.162
Posttreatment follow-up period, year, Mean±SD (range)	2.7±1.3 (1.5–4.5)	4.0±0.7 (2.5–4.5	3.7±1.1 (0.5–4.9)	0.071
Baseline ALT level (U/L)	81.4±24.9	164.2±82.0	107.5±77.3	0.063
Liver cirrhosis, n (%)	1 (20)	2 (20)	4 (6.1)	0.100
Positive HBeAg, n (%)	0 (0)	0 (0)	4 (6.1)	0.354
Baseline HBV DNA negativity, n (%)	3 (60)	7 (70)	24 (36.4)	0.032
Baseline HCV RNA level (log IU/ml)	5.5±1.1	5.8±1.1	5.4±1.5	0.748
Achievement of an HCV SVR, n (%)	3 (60)	7 (70)	49 (74.2)	0.658
HBV DNA negativity EOT, n (%)	3 (60)	8 (80)	36 (54.5)	0.138
HBV DNA negativity 6 months after EOT, n (%)	4 (80)	5 (50)	27 (40.9)	0.344
HBV DNA negativity 1 year after EOT, n/N (%)	5/5 (100)	5/7 (71.4)	16/53(30.2)	0.004

HBsAg, hepatitis B s antigen; SD, standard deviation; ALT, alanine aminotransferase; HBeAg, hepatitis B e antigen; HBV, hepatitis B virus; HCV, hepatitis C virus; SVR, sustained virologic response; EOT, end of treatment.

## Discussion

Loss of HBsAg is the ultimate endpoint of antiviral therapy for chronic HBV infection. It is accompanied by disease remission in terms of ALT normalization and by a significantly decreased risk of LC, hepatic failure, and HCC [22]. Spontaneous seroclearance of HBsAg is a rare event in CHB, with an estimated annual incidence of 0.1% to 0.8% [9]. HBsAg seroclearance is also observed in patients receiving treatment for CHB including nucleoside/nucleotide analogues and IFN. Several studies of lamivudine treatment reported absence or less than 2% HBsAg seroclearance [23], [24], [25]. One-year treatment with adefovir dipivoxil resulted in 1.6% HBsAg seroclearance, and a recent study using tenofovir disoproxil fumarate for 48 weeks resulted in 3% HBsAg seroclearance [26]. Another study reported a 5% HBsAg seroclearance rate after standard IFN-α treatment for 32 weeks [27]. A higher HBsAg seroclearance rate of 7–11% was reported after 1-year pegylated IFN treatment in patients with CHB monoinfection [21], [28].

Clinical and laboratory studies have shown that the HBV and HCV interact with each other and affect immune responses. Most dually infected cases have detectable levels of HCV viremia and very low values of serum HBV DNA [29], [30]. In cases of HCV superinfection with HBV, HBsAg seroclearance has been reported [31]. A large-scale prospective trial designed to evaluate the efficacy of pegylated IFN-α-2a plus ribavirin combination in HBV/HCV dually infected patients reported 11.2% HBsAg seroclearance [19]. The long-term data reported here show that 25.6% of HBV/HCV dually infected patients treated with IFN-α/ribavirin combination therapy achieved HBsAg seroclearance during the 5-year follow-up period. This higher rate of HBsAg seroclearance suggests that the viral interaction between HBV and HCV may influence the rate of HBsAg seroclearance.

Many factors are related to HBsAg seroclearance, including HBV genotype. Yuen et al. reported that HBsAg carriers with genotype B infection were more likely to clear HBsAg than those with genotype C infection [22]. Others reported higher HBsAg seroclearance in genotype A as compared to genotypes B, C, or D after pegylated IFN-α treatment in HBeAg-positive patients [21], [28], [32]. In HBe-negative patients, Marcellin et al. reported that HBsAg seroclearance did not appear to be genotype related [21]. In the present study, most of our patients were HBeAg-negative. However, the influence of HBV genotype was not evaluated due to limited serum sample availability.

HBV DNA is another factor associated with HBsAg seroclearance. Several studies reported that serum HBV DNA becomes undetectable 1 to 2 years after HBsAg seroclearance [22], [33]. In the present study, HBV DNA negativity 1 year after EOT was the most significant predictive factor of HBsAg seroclearance. We also found a 53.3% 5-year cumulative incidence of HBsAg seroclearance in patients with HBV DNA negativity 1 year after EOT. The findings implied that HBsAg seroclearance might be promoted by IFN/RBV in HBV/HCV dully-infected patients through the achievement of HBV DNA seroclearance.

Interestingly, we found 35.5% of patients who had HBV DNA negativity 6 months after EOT had reappearance of HBV DNA 1 year after EOT. The positive predictive value of HBsAg seroclearance in patients with HBV DNA negativity 6 months after EOT was only 25%. Using the corresponding SVR definition of HCV is, therefore, not suitable for HBV response, and cannot predict subsequent HBsAg seroclearance. Three of six (50%) patients who had detectable HBV DNA 6 months after EOT and became undetectable 1 year after EOT had HBsAg seroclearance in the long-term follow-up period. Based on this result, HBV DNA 1 year after EOT, instead of 6 months after EOT, might be recommended for predicting HBsAg seroclearance in clinical settings.

We also observed that liver cirrhosis was another predictive factor for HBsAg seroclearance. Increasing age has been associated with HBsAg seroclearance [34], [35]. In the current study, HBsAg seroclearance rate in older patients tended to be higher than that of their counterparts. A longer time period of infection, an older age and a lower HBV DNA level are at risk of cirrhosis and may largely contribute to this observation.

More recently, the low baseline levels of HBsAg was demonstrated to be significantly associated with HBsAg clearance 6 months after antiviral therapy in patients dually infected with HBV/HCV [20]. However, we did not quantify the levels of HBsAg due to no serum sample available.

HBsAg seroconversion is not infrequent in Caucasian patients undergoing IFN therapy [36], but is very rare in the Asian population [37]. A recent study demonstrated a 3.5% HBsAg seroconversion rate at 3 years post-treatment in HBeAg-negative patients treated with pegylated IFN-α-2a [21]. In the present study, the 5-year cumulative incidence of HBsAg seroconversion was 8.3%. The fact that a substantially higher rate of HBsAg seroconversion was found in our study as compared to studies of CHB monoinfection suggests that HCV superinfection may interact with HBV and enhance the appearance of anti-HBs after loss of HBsAg [38], [39].

In conclusion, we demonstrated that interferon-α/ribavirin had long-term effects on HBsAg seroclearance in dually HBV/HCV infected patients with a 5-year cumulative incidence of approximately 25%. Baseline cirrhosis and HBV DNA seronegativity 1 year after EOT were significant factors predicting HBsAg seroclearance. Those patients with sustained undetectable HBV DNA after combination therapy had a higher probability of late HBsAg seroclearance. The 5-year cumulative incidence of HBsAg seroconversion was 8.3%. HBV DNA negativity at baseline and one year after EOT had a trend for HBsAg seroconversion.
